# Mid-IR standoff measurement of ageing-related spectroscopic changes in bitumen in the 6 µm (1700 cm^−1^) region. Part 1: Measurement strategy and instrument design principles

**DOI:** 10.1038/s41598-025-01135-7

**Published:** 2025-07-11

**Authors:** Jane Hodgkinson, Nicholas M. Davis, Cormac Browne, Kamal Nesnas, Stuart McRobbie, Alex Wright, Ralph P. Tatam

**Affiliations:** 1 Engineering Photonics, Cranfield University, Cranfield, Bedfordshire MK43 0AL UK; 2 TRL, Crowthorne House, Nine Mile Ride, Wokingham, RG40 3GA UK; 3 Present Address: WSP, King James VI Business Centre, Friarton Road, Perth, PH2 8DY UK; 4 Present Address: National Highways, Safety, Engineering and Standards, Guildford, GU1 4LZ UK; 5 Present Address: XAIS-PTS, Unit 1 Rough Hey Road, Grimsargh, Preston, PR2 5AR England, UK

**Keywords:** Civil engineering, Infrared spectroscopy, Optical sensors

## Abstract

A strategy is described to make in-situ measurements of a spectroscopic marker of ageing in bitumen binders used on asphalt-paved roads. Oxidation of bitumen at the surface increases the number of carbonyl (C=O) bonds, and this can be measured in the 6 μm region (1700 cm^−1^) of the mid-infrared. A measurement strategy is proposed to make standoff measurements of surface reflectivity in this region, despite the challenge presented by numerous strong absorption lines from atmospheric water vapour within the optical path. An instrument design is described to make measurements at 4 discrete laser wavelengths, namely 1593.0, 1641.4 and 1731.3 cm^−1^ (around 6 µm) and at 2633.6 cm^−1^ (3.8 µm), the first 3 responding to carbonyl absorption and the fourth acting as a spectral reference that is substantially unaffected by ageing. Part 2 of this paper describes the implementation of such an instrument and its experimental performance.

## Introduction

**Fig. 1:**
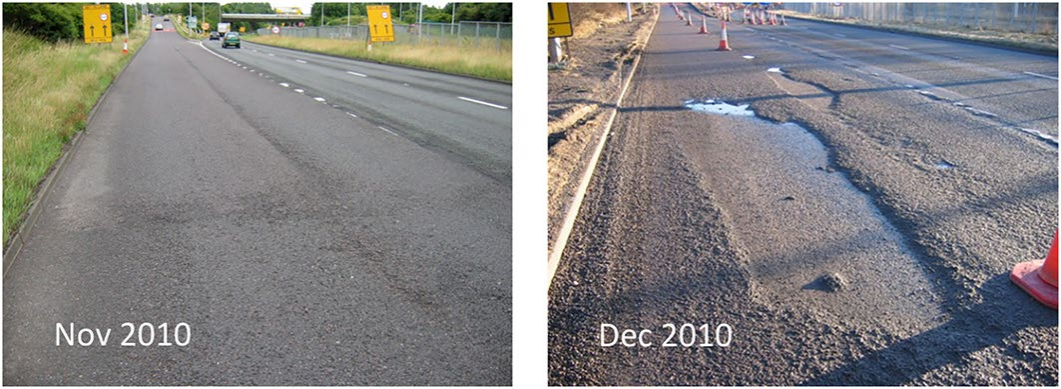
Strategic road network in England: Rapid deterioration in road surface as a result of fretting, over the course of 1 month.

Thin pavement surfacings have become increasingly prevalent in modern road construction. Their use limits road disruption during refurbishment and reduces the required quantity of primary aggregate. However, these pavements can suffer from rapid deterioration following traffic wear and environmental exposure (e.g., sunlight, salt and water), reducing their service life. A major problem is the onset of fretting, with little prior warning, whereby small aggregates at the surface lose adhesion with the bitumen binder. Serious potholes can result within 1–2 months via the accelerated loss of aggregate, as shown in Fig. 1.

Pavement monitoring typically relies on detection of mechanical behaviour (braking friction and surface movement under load) and surface defects (shape and visual appearance)^1^. Such physical parameters are surveyed at traffic speed (50mph or 20 m s^−1^) without closing roads. However, they can only identify severe defects that are clearly visible. Measurement of chemical degradation, which may precede physical changes, could enable defects to be identified earlier, but its study is in the early stages due to a lack of technology capable of surveying large lengths of road surfaces^2^.

Bitumen hardening, embrittlement and loss of adhesion contribute significantly to road surface failure and are linked to oxidation, which is considered the primary chemical degradation mechanism^3,4^. Other features of ageing are a loss of the (already low) volatile content and physical hardening as a result of molecular reorganisation, the latter being considered reversible, however the effects of these are limited compared to oxidation^4^.

The formation of carbonyl bonds (C=O) by oxidation of the hydrocarbons in bitumen is considered a universal marker of ageing for different bitumen types (including virgin, polymer- and sulfur-modified bitumen)^2^. Carbonyl bonds may be formed as ketones, carboxylic acids and dicarboxylic anhydrides, and any alkyl sulfurs present can also be oxidised to sulfoxides^3^. The process requires the diffusion of atmospheric oxygen (over several mm) and is promoted by photo-oxidation reactions resulting from UV exposure/penetration into the upper few µm of the surface^3^. Ageing may be accelerated for test purposes. The Thin Film Oven Test and Rolling Thin Film Oven Test (RTFO) are often used to study short-term ageing in transport and installation, whereas the Pressure Ageing Vessel (PAV) and high UV exposure simulate longer-term in-service ageing^3^.

Optical spectroscopy is well-suited to measure these chemical changes since the mid IR “fingerprint region” responds primarily to chemical bonding. Alternative methods to study bitumen ageing include atomic force microscopy, thin-layer chromatography, gel permeation chromatography and dynamic shear rheometry^3,5^, however none of these has the potential to be used as a non-destructive evaluation tool. Fluorescence microscopy has also been used to probe bitumen microstructure, but this also requires sample extraction and preparation.

Alternative spectroscopic approaches for standoff detection have been used in other fields, especially the detection of potentially hazardous materials such as explosives^6^, and identification of materials on production or recycling lines^7^. Methods include laser-induced breakdown spectroscopy (LIBS)^6,8^ and Raman spectroscopy^9^. Compared with IR absorption spectroscopy, neither has accumulated such a strong body of evidence associated with measurement of ageing of asphalt. Reports of their use in this application are therefore limited. LIBS is used to provide elemental analysis and as such would have problems associated with the presence of other oxygen-containing molecules (including gaseous O_2_). Caputo et al*.* have investigated the use of (laboratory-based) Raman spectroscopy to study asphaltenes extracted from aged and fresh bitumen^10^. They found that the spectra were “almost identical”, concluding that derived parameters “cannot be considered as good indicator of bitumen aging”.

Research using laboratory^2,5^ or hand-held^3^ FTIR spectrometers to measure both artificially and field- aged bitumen and asphalt has identified an ageing-correlated increase in the spectral absorption associated with carbonyl bonds in the 1760–1620 cm^−1^ region of the IR (around 6μm), indicative of oxidation^3^. Because of the strong absorption of atmospheric water vapour across this region, these methods require close physical contact with the sample (which can lead to bitumen sticking to the optics) or dry atmospheric purging, and the low spectral brightness of the source dictates long (1 min) averaging times. So the question is, can measurements be made at traffic speed in a standoff geometry?

Here, the scientific challenges inherent in building such an instrument have been considered. Starting with the information available in the mid-IR spectrum, this paper looks at how this might be measured in a non-contact, standoff geometry. Geometric constraints and potential noise sources are considered. The presence of atmospheric water vapour poses a serious challenge that, the work shows, can be addressed both by using narrow linewidth lasers and by providing an additional reference beam path that is also exposed to water vapour. This is a fundamental concern for any standoff measurement operating in the 5–7 µm region, which is consequently usually avoided if possible^11^. To the authors’ knowledge the problem has not been previously addressed, therefore its solution could open up the region for other applications. The effect of other atmospheric gases, which could also potentially interfere with the measurement, has been considered. Finally, potential noise sources and the instrument design needed to support the required signal:noise ratio are considered. Here, the most challenging aspect is the speed of the measurement required to support travel at up to 50mph (~ 20 m s^−1^), which requires spectral data to be taken quasi-simultaneously, within a timescale corresponding to the passage of granular material beneath the instrument (> 20 kHz). In Part 2, the development of such an instrument is reported, including experimental challenges and performance^12^.

## Spectroscopic basis for the measurement

Various groups have used different metrics to indicate ageing based on measurement of absorption peaks in FTIR spectra, some referring to these as a carbonyl index. Examples are shown in Table 1. Based on correlations with the physical symptoms of ageing, the chemical changes measured via FTIR are now considered to have potential to quantify ageing directly^13^. Because of the low transmission of bitumen, sample extraction is required or IR spectra can only be probed in reflection for bulk samples, with attenuated total reflectance (ATR) being the “method of choice” for the bulk^14^. Hofko et al. measured identical bitumens with FTIR-ATR and found that different analyses of the spectra produced different levels of repeatability^14^. They recommended use of an absolute baseline, integrated area measurements and peak referencing/normalisation options as shown in Table 1.

**Table 1:** Examples of FTIR analysis used to study the ageing of bitumen.

Ref	Sample ageing	Sample preparation	Measurement type	Peaks analysed
Wang et al*.*^5^	Field aged	Solvent extraction from a core sample, then centrifuge	Lab-based ATR	Area of 1700 cm^−1^ peak (C=O bonds) normalised by area of 1376 cm^−1^ peak (C–H bonds)
Wu et al.^15^	Field aged	Bitumen recovered from sample core (method not stated)	Lab-based, not stated (ATR?)	Area of peak at 1700 cm^−1^ normalised by Σ (areas of spectral bands between 4000 and 400 cm^−1^)
Lamontagne et al.^16^	Field aged and heated within custom FTIR oxidation cell	Solvent extraction and evaporation	Transmission through KBr plate	Area under 1753–1635 cm^−1^, normalised by area under other bands from 2983 to 724 cm^−1^
Arafat et al*.*^13^	RTFO and PAV	Solvent extraction and filtering in the field	Hand-held FTIR with ATR probe	Baseline-corrected peak heights at 1695 cm^−1^ (C=O) normalised by that at 2920 cm^−1^ (CH_2_)
Nivitha et al.^17^	RTFO and PAV	Solvent extraction and evaporation	Transmission through thin film on KBr plate	Area under 1768–1725 cm^−1^, normalised by Σ (area of several bands between 1720 and 715 cm^−1^)
Hofko et al.^14^	RTFO	Direct application of heated bitumen	Lab-based FTIR-ATR	Area under 1666–1746 cm^−1^ carbonyl region, normalised by region 2923 cm^−1^ (aliphatic) and referenced to area under region 1319–1520 cm^−1^ (also aliphatic)
Cheraghian et al*.*^18^	RTFO and UV	Not stated	Lab-based FTIR, transmission	Area under band at 1700 cm^−1^ normalised by Σ area under bands at 1460 and 1375 cm^−1^
Porot et al*.*^19^	RTFO and PAV	Not stated	11 lab intercomparison study: 9 used ATR, 2 used transmission	Area under different ranges from 1728 to 1674 cm^−1^, normalised by CH_2_/CH_3_ bands area under different ranges from 1510 to 1330 cm^−1^
Bowden et al.^3^	RTFO and UV	Close contact with unaltered sample	Lab-based and hand-held DRIFTS	Area under 1755 to 1636 cm^−1^

Table 1 reveals a limited scientific consensus on the precise definition of the carbonyl index, and this is also reflected in the laboratory intercomparison study of Porot et al*.*^19^. For transmission and ATR measurements, most groups use the area under an absorption band, establishing a baseline between adjacent minima, however the beginning and end of the band are a matter of choice. Hou et al. have reviewed the field^2^ and identified shifted peak positions of several different carbonyl bonds over the region 1620–1775 cm^−1^, corresponding to whether the C=O group is part of an amide, ketone, carboxylic acid, aldehyde or ester. Researchers therefore choose to measure peaks at different wavenumbers based on the presentation of their spectra. However, Yao et al*.* have shown that when looking for the signs of ageing, carboxylic acids (1700–1725 cm^−1^) and ketones (various types in the region 1665–1850 cm^−1^) showed the greatest increase^20^. Thus, spectral features associated with ageing can appear across the carbonyl region depending on the precise chemical makeup of the bitumen, but the strongest may be found in the 1665–1725 cm^−1^ region.

Other ageing-related binding changes can also be visible in FTIR spectra. For asphalts that contain sulfur, this may oxidise to sulfoxides which have been identified in the 1043–1010 cm^−1^ region^17,21^ and are used to establish a sulfoxide index. Changes may also be seen in some aromatic bands (1625–1535 cm^−1^) and aliphatic bands (3000–2800 cm^−1^ and 1510–1350 cm^−1^), used for aromaticity and aliphaticity indices respectively^17,21^. In this work, a decision was made to concentrate on the carbonyl band because oxidation would be expected to give rise to changes in this band for all asphalt types.

Bowden et al*.*^3^ have recently reported the use of diffuse reflectance infrared Fourier transform spectroscopy (DRIFTS), which is of more direct relevance to this work. For naturally aged asphalt, features associated with ageing were attributed to carbonyl (C=O stretch at 1730 cm^−1^) and sulfoxide (S=O stretch at 1155 cm^−1^). Other features visible were attributed to carbonates (${\text{C}}{\text{O}}_{3}^{{2}-}$ asymmetric stretch at 1532 cm^−1^ and overtone at 2512 cm^−1^) and aliphatics (C–H stretch at 2915 cm^−1^ and 2844 cm^−1^).

DRIFTS spectra can be complicated by a mixture of specular and diffuse reflection. Around absorption features, changes in the refractive index and therefore specular reflectivity lead to first derivative-like features that can be interpreted as positive and negative absorption around the band centre^22^. Mixed in with these features are mainly absorptive features resulting from diffuse reflection. If the full reflectance spectrum is acquired, then in principle these effects can be separated and the absorbance provided via a Kramers–Kronig transform, however for many real samples this is reported to result in unstable spectral baselines and distorted absorption features^22^.

**Fig. 2:**
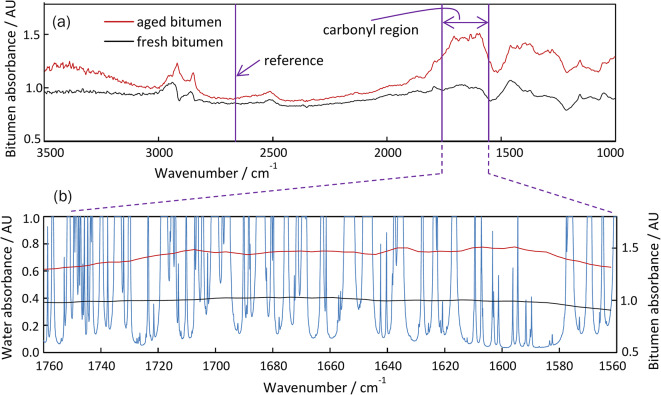
(**a**) Diffuse reflectance spectra measurement with a hand-held FTIR spectrometer, for aged and fresh bitumen within asphalt samples^25^. (**b**) Section of bitumen spectra overlaid on absorption spectrum for atmospheric water vapour, calculated from the PNNL database for an optical pathlength of 1m and 6.6%vol (worst-case abundance at 90% RH and 40 °C).

Since asphalt has a granular surface, containing rough aggregate interspersed with smoother bitumen, a blend of both specular and diffuse reflection is possible in any measurement that does not select a flat region of the target^3^. This characteristic makes it challenging to choose discrete wavelengths at which to measure the reflectance; ideally, a full spectrum would enable interpretation. However, the work also showed that naturally aged samples may present with a higher proportion of diffuse reflection (and a visually less shiny surface), with spectra somewhat easier to interpret, with features associated with ageing generally broader than would be the case for ATR measurements (see for example Fig. 2a). For analysis of asphalt, Bowden^3^ has also suggested that it is important to be able to separate out the absorption due to carbonates in the aggregate (around 1543 cm^−1^).

In transmission measurements, the absorbance *A*_*T*_ at a wavelength *λ* is defined as^23^:

1 $${A}_{T}\left(\lambda \right)=-\text{log}\left(\frac{{\Phi }_{T}\left(\lambda \right)}{{\Phi }_{0}\left(\lambda \right)}\right)= -\text{log}T\left(\lambda \right),$$

where Φ_0_ is the incident radiant flux, Φ_*T*_ is the transmitted radiant flux and *T* is the transmittance. *A* is unitless but often quantified in Absorbance Units (AU). For absorption by gases (considered in “Spectroscopic signature of gases in exhaust fumes” section), the following relationship applies:

2 $${A}_{T}\left(\lambda \right)=\varepsilon \left(\lambda \right){\ell}c,$$

where *ε* is the molecular absorptivity per unit partial pressure or concentration (e.g. units in cm^−1^ atm^−1^), ${\ell}$ is the optical pathlength (e.g. in cm) and *c* is the partial pressure or concentration (e.g. in atm). Note that *ε* can be provided using base *e* or base 10; we have used base 10 here throughout for compatibility with standard formats used in FTIR spectrometers.

For conventional FTIR measurements, normalisation features (insensitive to ageing) are needed to quantify the effective optical penetration into the bulk material (ATR) or the amount of material present in the analysis, especially when solvent extraction has been used in sample preparation. Many research groups normalise their spectra using the area under other features, for example representing C–H bonding, which can also show changes on ageing^3^. In FTIR spectra, it is also common practice to measure a “blank” sample, to establish the level of light transmission in the absence of any absorption, and to be able to calculate the absorption using Eq. (1).

For measurement of diffuse reflection, by analogy with Eq. (1), the absorbance is the log of the ratio of unabsorbed (reflected) to incident light. It is assumed that the surface is semi-infinite and therefore there is no light transmitted, so all unabsorbed light must be reflected (effectively true for asphalt of several mm thick). However, since the reflected light may be scattered over a full range of angles, complete measurement of reflected light requires an integrating sphere. In practice for field measurements with the hand-held FTIR, reflected light is measured over a wide range of angles and the result compared to a standard diffuse reflector, usually diffusely reflective gold. This practice assumes that the bi-directional reflectance distribution function (BRDF) of the two materials is comparable. The reflectance of such samples can vary; the calibration standard used in this work (Labsphere Infragold diffuse reflectance target^24^) had a validated reflectance of > 94% across the mid-IR, but other targets can show reflectance dropping below 90% and varying with angle of incidence and polarity. Diffuse gold can therefore show some deviation from perfectly Lambertian reflectance^24^. However, as long as absolute measures are not required, the method ensures reproducibility. The absorbance *A*_*R*_ for diffuse reflection can then be considered equivalent to^22^:

3 $${A}_{R}\left(\lambda \right)=-\text{log}\left(\frac{{\Phi }_{R}\left(\lambda \right)}{{\Phi }_{S}\left(\lambda \right)}\right)= -\text{log}R\left(\lambda \right),$$

where *Φ*_*R*_ is the measured reflected radiant flux, *Φ*_*S*_ is the measured radiant flux reflected by the standard, and *R* is the reflectance. If the reflectance of the standard is not equal to unity, a scale factor is required to compensate.

In hand-held FTIR using diffuse reflection, changes to the surface height and tilt, which might affect the bulk level of diffuse scattering, are controlled by fixing the sample geometry. The sample must be in close contact with the collection aperture and normal to the collection axis. For a continuous measurement of diffuse reflection, we do not have this facility. Additional normalisation issues for standoff measurements therefore include changes to surface height and tilt, plus changes to the overall transmission of the optics, some of which would be exposed to the elements, and changes in the level of absorption of the signal by water vapour. To compensate for geometric effects, this work used a reference wavelength that avoided strong absorption features in the bitumen spectrum. This wavelength measurement fulfils the role of the “blank” measurement in laboratory FTIR transmission and the reference standard in hand-held FTIR measurement of diffuse reflectance.

Figure 2a shows example spectra of aged and fresh material, replotted from data measured by Bowden^25^. Spectra were measured using a hand-held, low-resolution FTIR (Agilent Exoscan), which measures diffusely reflected light collected over a large numerical aperture (NA) (unstated) where the collection optics had close/direct contact with the sample. Samples consisted of Stone Mastic Asphalt (SMA) (United Asphalt) containing 40/60 penetration grade bitumen with hard stone aggregates including limestone and granite. The samples had a maximum aggregate size of 10 mm and a binder content of 6%. The asphalt slabs were 200 mm × 200 mm × 50 mm, approx. 4 kg in weight and they complied with materials specification EN 13108. The aged sample was naturally aged on a building roof (Crowthorne House, UK) for 24 months from 2015 to 2017^25^. At this latitude, we would expect the annual received UVA dose (320–400 nm) to be in the range 175–225 MJ m^−2^. That figure is based on measurements published by the UK Health Protection Agency between 1990 and 2008 at a site in Chilton, UK (approx. 21 km N and 36 km W of Crowthorne House), using a UV photodetector with response in the range 320–400 nm (Macam Photometrics SD-104A)^26^. The bitumen was bound with an aggregate as asphalt, and the measurement was located so as to target the bitumen binder within the samples. Because of the need to place the instrument in close contact with the sample, combined measurements of both aggregate and bitumen could not be made repeatably. Absorbance (as − log*R*) was defined by reference to measurements made using a diffuse gold standard accessory supplied with the instrument.

Figure 2a also shows the spectral region corresponding to the appearance of carbonyl bonds, and our reference wavelength. It was decided to avoid wavelengths corresponding to absorption by alkanes, as used in ATR and transmission measurements, since otherwise the presence of oily deposits on road surfaces might create errors. For diffuse measurements in a standoff geometry, the quantity of material is fixed and the primary concern for normalisation is to measure the total level of reflected light, in the absence of material absorption, which might change as a result of macroscopic surface shape, distance and tilt angle, or optical occlusion.

Figure 2b illustrates the presence of multiple water absorption lines in this region, which pose a problem of spectral interference and reduction of signal strength. Passage of the analysed light through the open air cannot easily be avoided in a standoff geometry intended for use on the open road. The atmosphere around the device is likely to contain significant levels of exhaust fumes, with high levels of water vapour and other gases. This is considered in greater detail in “Spectroscopic signature of gases in exhaust fumes” section.

## Operational context

Major roads such as those in England’s Strategic Road Network include motorways and large trunk roads where there is a speed limit of 70 mph (112 km h^−1^). In order to be able to survey such roads safely without closing them, survey vehicles must operate at up to 50mph (80 km h^−1^), or around 20 m s^−1^. For comparison and compatibility with existing road survey data, measurements can be averaged over 0.5 s, which corresponds to 10 m of road when operating at this speed.

**Fig. 3:**
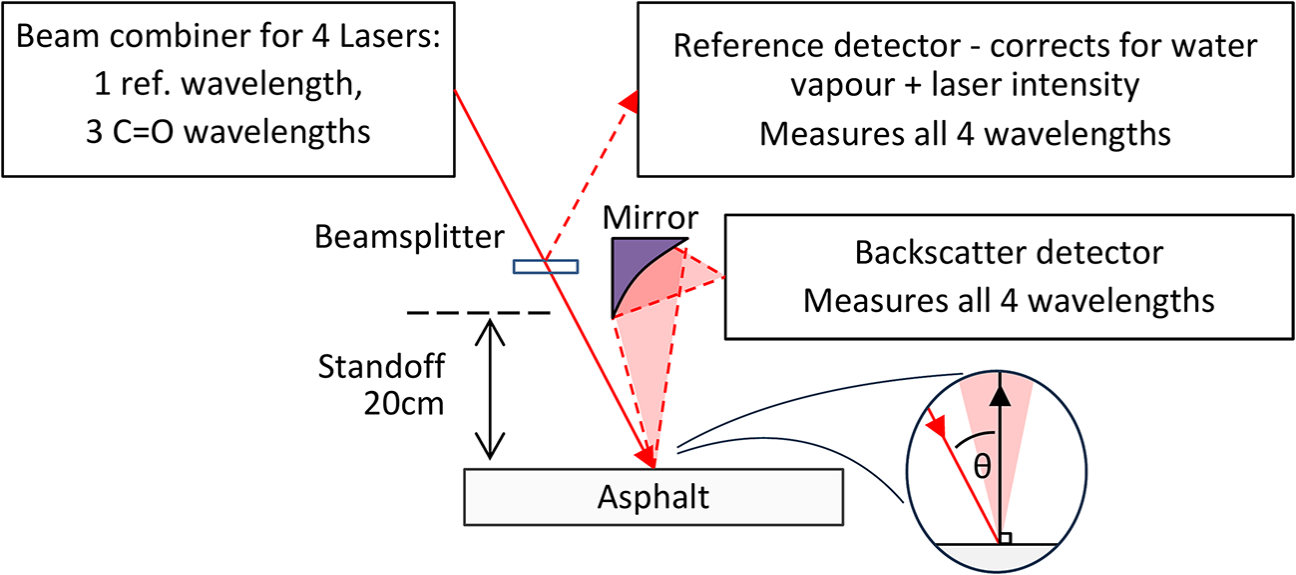
Measurement principle for standoff measurement of diffuse reflection at 4 wavelengths, 3 across the C=O band and one at a reference wavelength. Two detectors are used, one to measure the backscattered light at each wavelength, and a pathlength-matched reference detector to correct for laser output variation and residual water vapour absorption. Inset shows the angle of incidence, θ.

A working standoff of 20 cm between the pavement level and the lowest projecting item will allow movement of suspension and clearance of small foreign objects that may be present. The pavement surface cannot be assumed to be level; displacement from a planar surface can be caused by the operation of the vehicle’s suspension, a camber, rutting in wheel locations and compression or subsidence. The instrument must accommodate changes in surface height of ± 5 cm, reducing to ± 1 cm if a servo platform is used. In that case, a separate laser-based displacement sensor is used to measure the distance of the instrument from the surface, and a servo system is deployed to move a frame on which the instrument is mounted on the vehicle, so that the distance of the instrument from the surface is continuously maintained within the required range. The measurement geometry (see Fig. 3) must accommodate this while being suitable for spectroscopic measurement. Generally, the level of diffuse reflection from an illuminated spot on an ideally rough, Lambertian surface will be maximised for a collection axis that is normal to the surface. There is scope to vary the angle of incidence, within some practical constraints.

Spectral measurements need to be measured simultaneously (or quasi-simultaneously) over a surface consisting of a mixture of bitumen and aggregate. For asphalts applied as Thin Surfacing Systems on UK Strategic roads, the asphalt typically consists of a bituminous binder plus mineral aggregate of approximately 10–14 mm in size. Given the operational speed of 20 m s^−1^, this dictates that measurements at different wavelengths must be made within 1 mm of each other, or within 50 µs (at > 20 kHz) for a 10 mm aggregate, in order to “freeze out” any relative motion of the surface and provide stable spectral baselines. This timescale also has the benefit of reducing the effects of shock and vibration on measured spectra.

Based on the spectral data in Fig. 2, the instrument should be able to quantify the difference between absorbances of 0.1AU, leading to a requirement for a noise-equivalent absorbance of 0.01AU, corresponding to changes in reflected intensity of 2%. The mean reflectivity of bitumen can be as low as 2% (compared to diffusely reflecting gold) which demands an intensity precision of 4 × 10^–4^ (i.e. if the incident power is 1mW, the system must measure changes in reflected light of 0.4 µW). Because the absorbance of liquid (as opposed to vapour phase) water is strong and broad, the presence of liquid water on the surface must be avoided. It is typical for existing survey measurements to require dry conditions, so this is not a severe constraint.

The above operational constraints lead to a working specification for an instrument summarised in Table 2. Although such an instrument would be compatible with installation on a survey vehicle, for test purposes it was decided to implement the instrument on a trailer (with a shorter wheelbase, of 1m) to be pulled behind an existing survey vehicle.

**Table 2:** Working specification for instrument.

Parameter	Value	Comment
Minimum detectable absorbance, *A*_min_	0.01AU (1σ)	Difference after referencing; taken from spectra of aged versus fresh asphalt
Operating speed in traffic	20 m s^−1^ (20,000 mm s^−1^)	80 km h^−1^
Averaging time	Up to 0.5 s	Final provision of data over 10 m sections of pavement
Standoff distance	20 cm	
Variation in standoff distance	5 cm	Without servo control
	1 cm	With servo control
Variation in tilt angle	± 6°	For a 1m wheelbase, the angle for + 5 cm height difference on one wheel and − 5 cm on the other
Angle of incidence	15–45° incidence, 0° collection (diffuse)	Possible to reconfigure to determine best geometry experimentally
Relative humidity	0 to 90%RH	Not for use in rain; dry roads only

The chosen solution is shown schematically in Fig. 3. The collimated outputs from 4 lasers are co-aligned in an incident beam, a small proportion of which is split off to form a reference path while the remaining beam strikes the surface. A reference and backscatter detector each collect 4 signals, which can then be demodulated to provide wavelength separation. The rationale for this geometry and the use of referencing is described in later sections.

An instrument to measure asphalt ageing might be capable of deployment within an asset management system as follows. Measurement of a single absolute ageing indicator that can predict asset life, for every type of asphalt, is not considered practical. Asphalts have significant levels of variation between different types and different batches of the same type. Some types of bitumen have elevated levels of carbonyl bonding even prior to ageing. In addition, bitumen experiences short-term ageing (including oxidation) during handling and transportation, prior to paving.

It is therefore envisaged that an instrument would be used to measure changes that are associated with ageing, relative to a baseline measurement taken immediately after paving. It would be important to gain experience to link the age-related spectroscopic measurements to real-world asphalt performance and failure, by taking measurements alongside existing monitoring methods and at times of remediation where asphalt has failed. Ultimately, what is needed is a measurement that relates to the remaining asset lifetime that can be used as part of a predictive maintenance strategy. This may require development of a record of measurements over time, to establish the rate of ageing. Each asphalt type will no doubt age differently and will have a different relationship between its spectroscopic measurement and its remaining lifetime. This can be managed using existing records of the type and construction of asphalt used on each section of the road network.

### Spectroscopic signature of gases in exhaust fumes

A remaining concern is the potential presence of optical absorption by the atmosphere between the instrument and the asphalt pavement. For use in normal traffic on open roads, we considered the likely worst-case concentrations of gases, especially in the exhaust from road vehicles, listed in Table 3. The probable total gas pathlength in air was estimated to be 1 m. To have no significant effect on the *A*_min_ of 0.01AU, gases need to have a value of *εc* (in Eq. (2)) no higher than 10^–4^ cm^−1^. Gas absorption was calculated using data from the PNNL^27^ and HITRAN^28^ databases.

**Table 3:** Gases present in exhaust fumes, their likely worst-case concentrations and level of absorbance over a 1m pathlength.

Gas species	Worst-case concentration	Reason/reference	Max absorbance/AU	
			Ref region^a^	C=O region^b^
Water, H_2_O	6.6%	90%RH at 40 °C	1.8 × 10^–3^	**65**
Carbon dioxide, CO_2_	1%	Mixed exhaust/air, TRL data	4.1 × 10^–4^	< 6.2 × 10^–4^
Carbon monoxide, CO	20 ppm	Worst-case 8 h exposure limit^29^. UK consistently below 10 μg m^−3 ^^30^	–	–
Nitrogen dioxide, NO_2_	0.5 ppm	Worst-case hourly average can be 700 μg m^−3^^31^, or 0.34 ppm	–	1.4 × 10^–3^
Nitric oxide, NO	500 ppm (4000 ppm occasionally)	TRL Euro V and Euro VI truck data	–	3.9 × 10^–4^ (0.0031 occasionally)
Ammonia, NH_3_	100 ppm (900 ppm occasionally)	TRL Euro V and Euro VI truck data	3.6 × 10^–5^	**0.035 (0.32 occasionally)**
Ozone, O_3_	10 ppb	8-h running mean is below 8μg m^−3 ^^30^, corresponding to 2.3ppb		
Sulfur dioxide, SO_2_	2 ppb	Annual mean concentrations below 5 μg m^−3 ^^30^, corresponding to 1.75ppb	–	–
Methane, CH_4_	100 ppm	Large outdoor natural gas leak, inferred from^32^	1.3 × 10^–3^	2.7 × 10^–4^
Ethane, C_2_H_6_	10 ppm	As above: max 10% of ethane	2.9 × 10^–5^	–
Propane, C_3_H_8_	3 ppm	As above, also present in calor gas	1.1 × 10^–5^	–
Butane, C_4_H_10_	2 ppm	As above, also present in calor gas	9.3 × 10^–6^	–
Pentane, C_5_H_12_	1 ppm	As above	6.4 × 10^–6^	–
Octane, C_8_H_18_	100 ppm	Present in petrol, also indicative of other medium hydrocarbons	1.3 × 10^–3^	1.4 × 10^–4^
Methanol, CH_3_OH	100 ppm	Common solvent, indicative hydrocarbon	2.6 × 10^–4^	2.9 × 10^–4^
Benzene, C_6_H_12_	2 ppb	UK meets the limit value of 5 μg m^−3 ^^30^, corresponding to 1.4 ppb	–	–

Bold indicates potential for spectral interference above the required noise-equivalent absorbance.

^a^Region 2700–2600 cm^−1^ (3.7–3.85 µm).

^b^Region 1760–1590 cm^−1^ (5.7–6.3 µm).

There were no significant spectral interferences from gases present in car exhausts in the chosen reference region. Within the C=O region, ammonia has the potential to give spurious absorption signals when levels occasionally rise above 100 ppm, whereas water vapour has the potential to reduce reflected radiant fluxes significantly, with problems for availability of the measurement. As Fig. 2 shows, even in the trough regions between absorption lines, water absorption is significant; a factor of around 20–30 greater than the level at which there would be no problem, for a worst-case scenario of saturated water vapour at 40 °C.

The consequences of this are that measurements should be made only in these “water windows”, i.e. narrow regions of light transmission between the numerous absorption peaks of water, and that further techniques will be required to remove the effects of water vapour absorption from the measurement. The following options are possible:

(i) Purge the optical path using dry air, as has been done successfully for lab-based measurements. However, this would require a form-fitting flexible skirt around the entire optical path, in contact with the ground while moving at speed, and considerable quantities of dry air for purging (estimated at tens of litres/min if a well-sealed skirt could be engineered). Removing water vapour from the air supply at high flow rates would also be a challenge, or considerable storage capacity would be needed for dry air.
(ii) Use a reference beam with matched pathlength to automatically correct the measurements, assuming that water vapour is well-mixed between the measurement path and reference path.
(iii) Measure the water vapour content and correct measurements accordingly. This would need to be done at high speed, matching the rate of data collection for spectroscopic measurements.

The favoured option was (ii), which is analysed in “Water windows and final choice of lasers” section and described in Part 2^12^. The possible pathlength mismatch between the reference and measurements paths is fundamentally dictated by the extent of variation in standoff distance of ± 1 cm, reducing the problem of water absorption over a 1 m pathlength to the length of the unmatched pathlength. For an angle of incidence θ, the potentially uncompensated pathlength of the incident beam is ± 1 cm/cosθ, and the uncompensated pathlength of the collection beam (which is normal to the surface) is an additional ± 1 cm (see Fig. 5). For θ = 30°, the total equates to 2.2 cm.

## Measurement principles

The operational needs of Table 2 translate into the following technical constraints and requirements:

- High spectral brightness source (laser based, not thermal).
- Narrow spectral resolution, ideally < 0.3 cm^−1^, to operate within water vapour windows.
- Quasi-simultaneous acquisition of spectra within 50 µs (20 kHz).

The requirement to measure spectra within 50 µs eliminates many potential methods of measuring full spectra. Conventional FTIR instruments, requiring physical movement of an interferometer arm in order to measure a complete spectrum, are orders of magnitude away from taking a full spectrum within this timescale.

Measurement using a grating spectrometer in combination with a diode array sensor would be possible, since spectra would be acquired simultaneously, and the required resolution could be obtained with a suitable choice of grating. However, the standoff distance also dictates a bright light source, which with this type of spectrometer would require a superluminescent source. Operation within the 5–7 μm region is problematic. Not only does the presence of water vapour create measurement problems, but also those problems mean that the region is often avoided by developers, if possible. Consequently the market for technology in this region, and technology availability, is limited because of generally low demand. At the beginning of this project, commercially available superluminescent sources had not been developed for the 6 µm region. A further issue for such a solution would be the narrow aperture normally required for a grating spectrometer, which would significantly reduce throughput for a diffusely reflected beam. Tunable filters might be used instead, but the speed requirement again rules out available technology. Dual frequency-comb systems were also considered. At the start of this work, they were also not developed for this region in a package that could be field-deployed, but turnkey systems are now available in a form that might be adapted to field use^33^.

External cavity quantum cascade lasers (EC-QCLs) can be tuned widely (≥ 100 cm^−1^), have the required wavelength coverage for the C=O region, and in some configurations they have suitably narrow linewidths. Recently, Vanier et al*.* have used a widely tunable EC-QCL covering the wavelength region 745 to 1920 cm^−1^ (5.2 µm to 13.4 µm) for identification of minerals with a 100mm standoff^34^. High quality measurements were possible, however the linewidth of the pulsed source (2 cm^−1^) meant that operation within the windows of atmospheric water vapour was not possible.

Different EC-QCLs with rapid scanning are compared in Table 4. Note that the above requirements translate to a scanning speed of 3 × 10^6^ cm^−1^ s^−1^. The system developed by Lyakh et al*.* has the potential to be suitably rugged for field use, since the QCL is tuned with an acousto-optic modulator (AOM) rather than by using moving parts (which is also the key to its high speed operation)^42^. However, broad linewidths of 1.5 or 4.7 cm^−1^ would be incapable of resolving spectra within the water windows in our region of operation.

**Table 4:** Comparison of mid-IR laser solutions for rapid acquisition of spectra, showing different combinations of wavelength range, acquisition frequency and linewidth.

Laser type	Wavelength coverage	Scan frequency/kHz	Scan speed/cm^−1^/s	Linewidth/resolution (typical)	Reference
EC-QCL, pulsed	1370 to 1850 cm^−1^ (or wider)	Not stated	2.5 × 10^4^	2 cm^−1^	Block Engineering Lasertune^35^
EC-QCL, pulsed	4–13 μm	Not stated	5 × 10^3^	1 cm^−1^	Daylight Solutions Hedgehog^36^
EC-QCL, CW	30 cm^−1^ mode-hop free	Not stated	5 × 10^3^	100 MHz (0.003 cm^−1^)	Daylight Solutions Hedgehog^36^
EC-QCL, square-wave modulated	1520 to 1625 cm^−1^	200 Hz	2 × 10^4^	0.2 cm^−1^	Brumfield et al.^37^
EC-QCL with rotating polygon mirror	1520 to 1625 cm^−1^	4 kHz	4.2 × 10^5^	0.05 cm^−1^, mode spacing 0.5 cm^−1^	Revin et al.^38^
EC-QCL	7cm^−1^ at 992 cm^−1^	1 kHz	7 × 10^3^	0.38 cm^−1^	Tsai and Wysocki^39^
EC-QCL, pulsed	1090 to 1390 cm^−1^	6 kHz	1.8 × 10^6^	1 cm^−1^	Hugger et al*.*^40^
EC-QCL, CW	2105 to 2240 cm^−1^	100 Hz	13.5 × 10^3^	0.2 cm^−1^	Jia et al.^41^
EC-QCL, CW with AOM	1070 to 1135 cm^−1^	62.5 kHz	4.1 × 10^6^	1.5 cm^−1^	Lyakh et al.^42^
EC-QCL, quasi-CW with AOM	1020 to 1170 cm^−1^	62.5 kHz	9.4 × 10^6^	4.7 cm^−1^	Lyakh et al.^42^

The use of discrete lasers to measure at fixed wavelengths was therefore the only option available. This has the disadvantage of providing limited information, and preventing the measurement of full spectra, which would be easier to interpret especially given the difficulty of interpretation for diffuse spectra already discussed. However, discrete devices have the advantage of narrow linewidths, high brightness and fast measurement. It was considered essential in this work to explore the principle of making rapid measurements within the water windows in the region of interest, and to evaluate the pathlength referencing method used to mitigate the residual level of absorption by water vapour within the spectral troughs. Using a small number (3) of discrete wavelength QCLs allows completion of this investigation.

Technology exists to combine multiple discrete wavelength QCLs into a single package^43,44^, to provide increased spectral information (albeit, this has been developed to cover different wavelength ranges). Therefore, extension to a greater number of discrete measurements might be possible in future.

### Choice of operating wavelengths

It was decided to make measurements across the carbonyl region (1700 cm^−1^, 6 μm) because most types of asphalt would be expected to exhibit ageing-related spectroscopic changes in this region. It is possible that there might be some overlap with aromatic features visible in some FTIR-ATR spectra in the 1625–1535 cm^−1^ band; such features can also undergo ageing-related changes. A reference measurement was also proposed to fulfil the role of the spectroscopic “blank”, at a region (2600 cm^−1^, 3.8 μm) that we expect to be unaffected by ageing and to be generally free from strong absorption features, for example from aliphatic species. The reference wavelength would also need to be free from absorption by atmospheric gases (for example there is a strong absorption band of CO_2_ around 2400 cm^−1^ (4.2 μm)).

In this work, results have not been normalised for the amount of bitumen visible to the instrument by measuring at wavelengths associated with absorption by aliphatic species, a method used by several groups listed in Table 1. For on-road measurements, the presence of hydrocarbons for example from spilt oil could interfere with such a measurement and further work will be needed to establish a normalisation strategy. It is possible that a better understanding of the fraction of bitumen versus aggregate presented at the surface (and its influence on normalisation) could be obtained by combining with data from existing road surveys (surface profile, visible light imaging).

To the author’s knowledge, the challenge of atmospheric water in standoff measurements in the 5–7 µm region has not previously been addressed. Yang et al. developed a laser induced breakdown spectrometer operating in the mid IR, covering this region, but required inert gas purging of the optical path to the spectrometer^45^. Purging is also widely used in laboratory FTIR spectrometers used to measure ATR and transmission spectra to quantify the carbonyl index. In hand-held FTIR spectrometers, the problem is solved by requiring close physical contact with the sample and an enclosed optical path within the instrument. Here, a new approach is proposed, based on narrow linewidth lasers that can probe between water absorption lines.

### Water windows and final choice of lasers

The width of available water windows across the C=O region was the primary constraint on laser choice. It was decided to use discrete quantum cascade lasers (QCLs), which were commercially available for most water windows, and which had linewidths much narrower than the water windows.

**Fig. 4:**
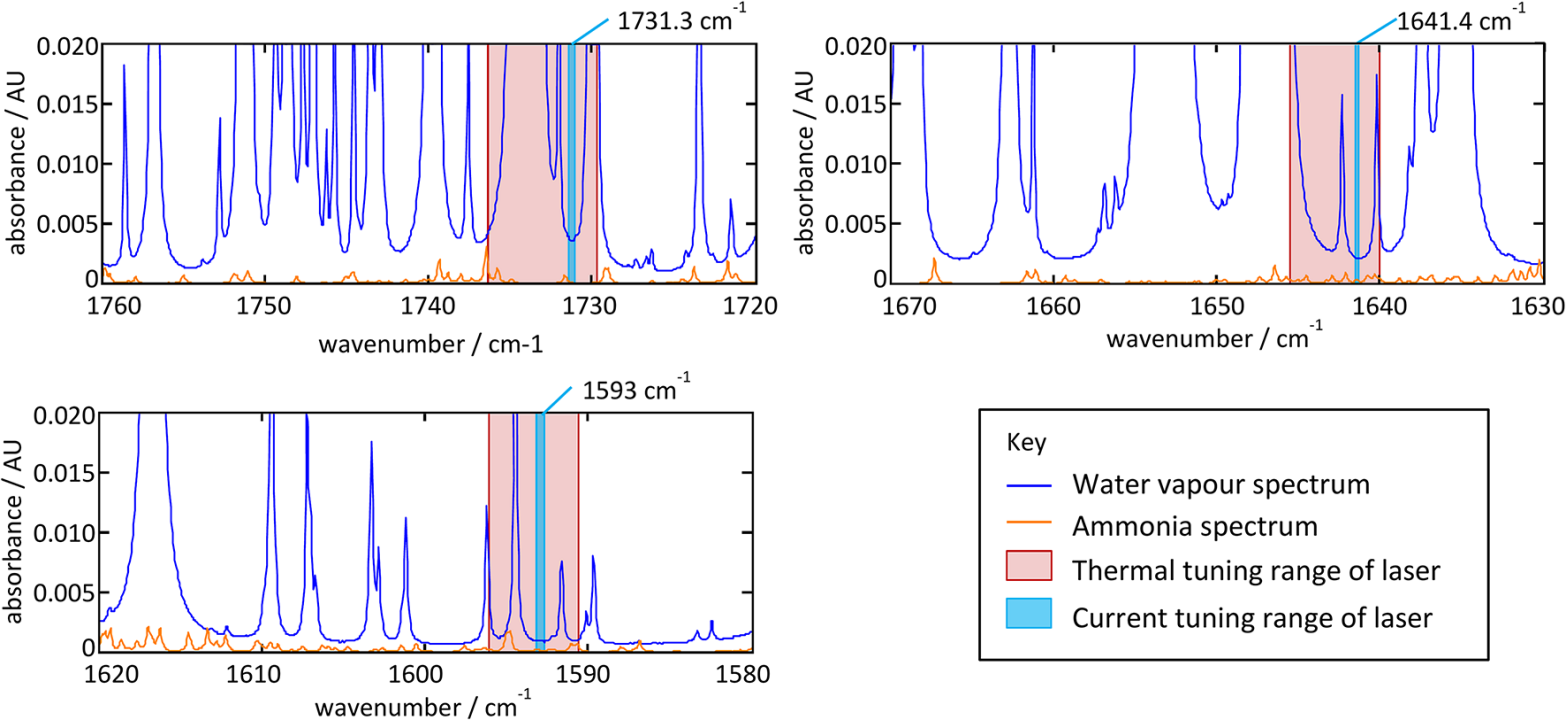
Tuning ranges for the three lasers chosen to span the carbonyl region, overlaid onto spectra for water vapour (6%vol) and ammonia (900 ppm) for a pathlength difference of 2.2 cm. The thermal and current tuning ranges show the full range of wavelengths accessible to each laser, and range corresponding to rapid injection current modulation, respectively. Y-axis absorbance figures translate directly into uncertainty in the measurement of asphalt.

To choose suitable discrete wavelength lasers, three favoured wavelength zones were chosen based on data captured using the hand-held FTIR (and shown in Fig. 4). Within these zones, the alignment of suitable water windows and laser availability resulted in a final choice of lasers whose output could be fine-tuned to fit within the identified water windows. A fourth laser was chosen at a reference wavelength; because spectral cross-response in this region was not problematic, there was greater tolerance on the precise choice of wavelength.

The final choice of lasers is summarised in Table 5. The expected tuning range of each laser, location of the measurement and water spectrum are shown in Fig. 4. For each laser, Fig. 4 shows the expected worst-case absorption resulting from water vapour (at 6% vol) and ammonia (at 900 ppm) for the possible mismatch in pathlength between the measurement and reference beams (caused by the potential variation in surface height of the road). Spectra were taken from the PNNL database^27^, where they are normalised to a concentration pathlength product of 1 ppm m, then re-scaled for the worst-case pathlength mismatch and gas concentrations. Although this doesn’t take full account of minor changes to spectra resulting from the need for different line broadening coefficients at different concentrations, it was considered close enough to provide a worst-case analysis of the positions and depth of spectral windows.

**Table 5:** Laser choices for reference and measurement.

Purpose	Wavenumber/cm^−1^	Wavelength/μm	Technology
Carbonyl	1593.0	6.2775	QCL
Carbonyl	1641.4	6.0924	QCL
Carbonyl	1731.3	5.7760	QCL
Reference	2633.6	3.7970	ICL

The pathlength mismatch of 2.2 cm is calculated here to be 1.2 cm for the incident beam (at 30°) plus 1 cm for the return beam (at 0°), given a servo-limited height variation of 1 cm. So long as the worst-case absorbance of these gases remains below the required limit of detection of 0.01AU, the pathlength-referenced measurement should not be disturbed by the presence of either gas. Further detail on laser characteristics and tuning ranges is given in Part 2 of this paper.

Figure 4 reveals that even within the water windows, there is a small residual level of water absorption at each chosen wavelength. While the level of absorption is small (and would not result in a saturated signal), variations in this residual level still have the potential to disturb the measurement. Figure 4 allows us to estimate the effect on the measurement of changes in the concentration of water vapour between a worst-case minimum (assumed close to zero RH at low temperature) and maximum (assumed 90% RH at 40 °C in warm, saturated car exhaust). Exhaust gases exit vehicles while still warm and contain significant (potentially near saturated) levels of water vapour. On a cold, dry day, this could lead to significant variation in the concentration of water vapour within the measurement averaging period of 0.5 s. Within the chosen water windows, Fig. 4 shows that the potential uncertainty in the measurement that this might cause would be well below the required noise-equivalent absorbance of 0.01AU. Indeed, there would be scope to relax the constraint of ± 1 cm height variation within these windows. The level of absorption by ammonia would be well-corrected over virtually the entire wavelength range of interest.

This correction assumes that the level of water vapour in the reference beam path would be fully representative of that in the measurement beam path. However, in practice the two beams must be separated because one (the measurement beam) must strike the asphalt surface and the other (the reference beam) must not, and indeed it must remain at or above the standoff distance since the optics are above that level. If this condition is not met, the uncertainty imposed by variable levels of water vapour would be too high, and the instrument performance would be dictated by the degree of air mixing between the two beams.

## Critical issues and design rationale

### Laser modulation and demodulation

It was decided to detect modulated signals from each laser, in order to reject changes in ambient light levels and to enable separation of signals from each of the 4 lasers while using a single photodetector. Each of the 4 lasers would have its injection current sine-wave modulated at a different frequency *f* within the range 20–40 kHz, thus producing a modulated intensity for detection. Demodulation would then be completed at 1*f* using a series of lock-in amplifiers, 4 for each of the two detectors.

The choice of modulation frequency *f* was determined by the speed of operation of the instrument on the road (20 m s^−1^) and the need to produce spectral measurements that were quasi-simultaneous within 20 kHz. Thus, the lowest value of *f* could be 20 kHz and the highest no more than twice that, to avoid cross-talk of demodulation at harmonics of 2*f* or higher. Averaging of demodulated signals would be possible with integration time constants of no greater than 0.5 s, to allow averaging over road distances of 10 m at 20 m s^−1^. Shorter averaging periods by the lock-in amplifiers would allow rejection of transient signals (for example from road markings, detritus or manhole covers).

Modulation of the injection current at a frequency *f* creates a modulation in the output wavelength as well as in its intensity. Thus, the width of the water windows would constrain the amplitude of the injection current modulation and thereby intensity modulation of the output. If the modulation were to exceed this constraint, absorption by water would result in additional modulation of the measured signal, primarily at 2*f* but potentially with a 1*f* component, which would disturb the measurement.

### Standoff geometry

#### Diffuse reflection model

**Fig. 5:**
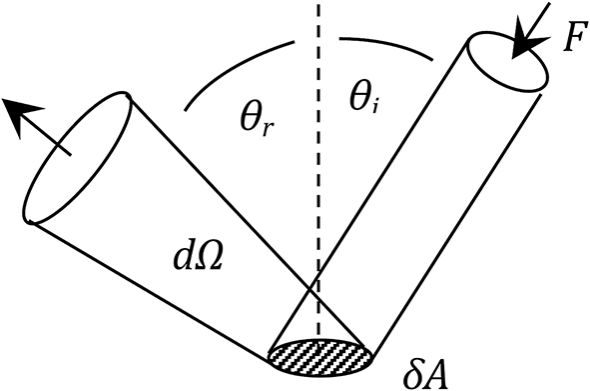
Geometric definitions used to describe diffuse reflection.

Consider a light beam incident on an area δA, as shown in Fig. 5. In cylindrical polar coordinates the incident beam has angle *θ*_*i*_ to the normal and rotational angle *φ*_*i*_ about the normal axis. For simplicity, the angle *φ* has been omitted from the figure, and we assume no dependence on *φ*.

The following radiometric definitions are given by Rees^46^. Let *F* be the flux density of incoming radiation in W m^−2^. The irradiance at the surface, *E*, is given by

4 $$E = F\hspace{0.33em}{\text{cos}}\mathit{\theta }_{i}\hspace{0.33em} {\text{W}}{\text{ m}}^{-{2}}.$$

*L*_*r*_(*θ*_*r*_*,φ*_*r*_) is defined as the radiance of scattered radiation in the direction *(θ*_*r*_*,φ*_*r*_), in W m^−2^ sr^−1^. (Note: in Fig. 5, *φ*_*r*_ is equivalent to *θ*_*r*_, but at right angles, i.e. out of the page.) Assuming no significant surface texture, the angular dependencies with *θ*_*r*_ and *φ*_*r*_ are similar, so we can set *φ*_*r*_ = 0 without loss of generality. The bidirectional reflectance distribution function, BRDF, is then defined as

5 $$BRDF = \frac{{L}_{r}\left({\theta }_{r}\right)\hspace{0.33em}}{E}\hspace{0.33em}{\text{s}}{\text{r}}^{-1}.$$

For a Lambertian surface, *BRDF* = 1/π, but this model was altered by Minnaert^47^ as follows,

6 $$BRDF = {\left({\text{cos}}\mathit{\theta }_{i}\cdot {\text{cos}}\mathit{\theta }_{r}\right)}^{k-1}\hspace{0.33em}\cdot \hspace{0.33em}{\text{constant}}{\hspace{0.33em}}\cdot \text{ s}{\text{r}}^{-{1}},$$

where *k* is the so-called Minnaert constant, a parameter that describes non-Lambertian scattering distributions (*k* = 1 for a Lambertian surface). With knowledge of the BRDF for asphalt, it would be possible to use the Minnaert model (or other more sophisticated models), however in the absence of this information the analysis proceeds by assuming Lambertian scattering.

The contribution to radiant flux dΦ in direction *θ*_*r*_, into solid angle *d*Ω, is given by

7 $${\text{d}}\Phi = L\;\cos \theta_{r} \;\;{\text{d}}A\;{\text{d}}\Omega \,{\text{W}}.$$

Now these definitions are applied to the proposed system. A small-angle approximation is made, and d*A* is defined as the projected cross-sectional area of the laser beam at angle *θ*_*i*_, then Eq. (4) gives

8 $$E = F\hspace{0.33em}{\text{cos}}\mathit{\theta }_{i} = \frac{{P}_{i}}{dA} \text{  W }{\text{m}}^{-{2}},$$

where *P*_*i*_ is the incident light power in W. The backscattered radiance from area d*A* is given by

9 $${L}_{r} = BRDF\cdot E = BRDF\cdot \frac{{P}_{i}}{dA} \hspace{1em}\text{W }{\text{m}}^{-{2}}\text{ s}{\text{r}}^{-{1}}$$

Now using Eqs. (7) and (9), we find the contribution to the received light power d*P*_*r*_, assuming that light is collected from the same illuminated area d*A* (which is true for the proposed system in which the laser beam underfills the detectable area):

10 $$\text{d}{P}_{r} = BRDF\cdot {P}_{i}{\text{cos}}\mathit{\theta }_{r}\hspace{0.33em}\text{d}\Omega \hspace{0.33em}\text{ W.}$$

Substituting for the BRDF with a Lambertian surface gives the following,

11 $$\text{d}{P}_{r} = \frac{1}{\pi }\hspace{0.33em}\cdot \hspace{0.33em}{P}_{i}\text{cos}\mathit{\theta }_{r}\hspace{0.33em} \text{d}\Omega \text{ W.}$$

**Fig. 6:**
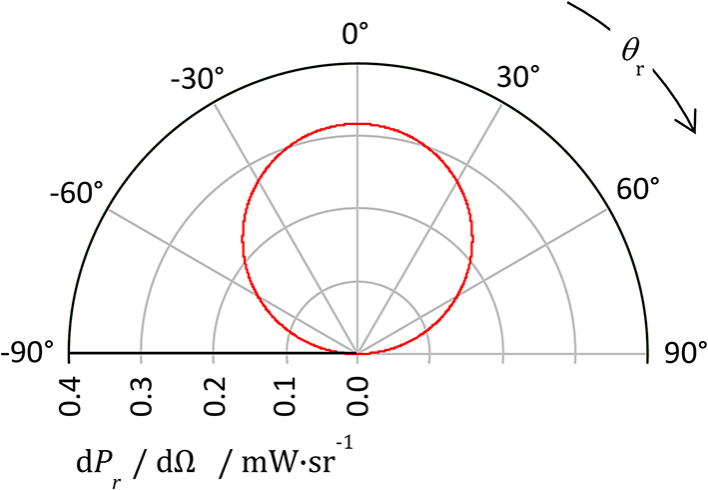
Level of light reflected from a perfectly Lambertian surface *P*_r_ per unit solid angle Ω (as d*P*_r_/dΩ) as a function of collection angle *θ*, for an incident beam of power 1 mW.

This is illustrated in Fig. 6. Clearly, for such a surface the signal to noise ratio will be maximised by a collection axis that is normal to the surface. And for an ideally Lambertian surface there is no dependence of the collected light on the angle of incidence *θ*_*i*_. The main purpose of altering the angle of incidence therefore would be to control the direction of specular reflection, either to deliberately collect it or to deliberately avoid it.

If a collection lens is centred at *θ* = 0, with diameter *a* at a distance *d* from the reflective surface, the solid angle subtended by the collection lens is given by

12 $$\Omega =2\pi \left(1-{\text{cos}} {\theta }_{col}\right) {\text{sr,  where }} {\theta }_{col}=\text{atan}\left(\frac{a}{2d}\right),$$

where *θ*_col_ is the angle subtended by the collection lens. Then the collected light power is given by

13 $$\frac{\text{d}{P}_{r} }{{\text{d}}\theta }= \frac{1}{\pi }\cdot {P}_{i}{\text{cos}}\theta\, \frac{\text{d} }\Omega{{\text{d}}\theta } = \frac{1}{\pi }\cdot {P}_{i}{\text{cos}}\theta \cdot 2\pi {\text{sin}}\theta ,$$

14 $${P}_{r} = 2{P}_{i}\hspace{0.33em} \underset{0}{\overset{{\theta }_{col}}{\int }}{\text{cos}}\theta \cdot {\text{sin}}\theta\, d\theta \, \text{W,}$$

Which evaluates to

15 $${P}_{r} = {P}_{i}\, {\text{sin}}^{2}{\theta }_{col}\, \text{W.}$$

#### Collection lens

From Table 2, the following basic geometric parameters are assumed: a standoff height of 20 cm, angle of incidence in the range 15°–45° and variation in standoff height of between 5 and 1 cm, depending on whether a servo is used. For modelling purposes, it was assumed that each beam might have a sine-wave modulated output of 1 mW peak-peak. A sine-wave modulation is straightforward to drive and demodulate, and 1 mW is a reasonable lower estimate of the modulated power achievable for a typical mid-IR laser. Also assumed was a high quality, electrically cooled (2-stage Peltier) detector with a detectivity D* of 5 × 10^8^ cm Hz^1/2^ W^−1 ^^48^.

Tables 6 and 7 summarise the light budgets for specular and diffuse reflections, respectively, as light makes its way through the system. The starting point is a sine-wave modulated emission of 1 mW (peak-peak) from each laser, which is considered an achievable level of power. The precise figure will depend on the individual laser and extent to which its output can be modulated and still remain within its water window. Other assumptions are the use of reflective optics with a mean reflectivity of 95% and 4 points of reflection, and a mean reflectivity of asphalt of around 2.5% (taking the values for aged asphalt in Fig. 2 would give around 3%). The results of the calculation in Table 6 show that specularly reflected light will be easily captured and detected by a high quality, TEC-cooled mid-IR detector, with a high signal to noise ratio of around 500. There is no requirement for a large lens, since the detected laser beam is narrow, as long as this is properly aligned (but see “Parallax management” section concerning parallax).

**Table 6:** Light budget for measurement of specular reflections at 30° angle of incidence.

Parameter	Proportion retained at each stage	Light power remaining/W	Comment
Light emitted from laser, after collimation	100%	3.5 × 10^–4^	Modulated emission of 1mW peak-peak: RMS value for sine-wave modulation
Light retained in beam-combining optics	86%	3.0 × 10^–4^	Using reflective optics with reflectivity ~ 95% for 3 reflections
Light retained in specular reflection	∥ 3.2% ⊥ 7.1%	∥ 9.7 × 10^–6^ ⊥ 2.2 × 10^–5^	Mean (low level) reflectivity of bitumen
Change in reflected power for a change in *A* of 0.01AU	2.3%	∥ 2.2 × 10^–7^ ⊥ 5.0 × 10^–7^	Equation (3)
Noise equivalent power (NEP) of detector		2.8 × 10^–10^	D* 5 × 10^8^ cm Hz^1/2^ W^−1^, 2 mm × 2 mm^48^, integration period 0.5 s

**Table 7:** Light budget for measurement of diffuse reflections.

Parameter	Proportion retained	Light power remaining/W	Comment
Light emitted from laser, after collimation	100%	3.5 × 10^–4^	Modulated emission 1 mW peak-peak: RMS value for sine-wave modulation
Light retained in beam-combining optics	86%	3.0 × 10^–4^	Using reflective optics with reflectivity ~ 95% for 3 reflections
Light retained in Lambertian reflection (all angles)	2.5%	7.6 × 10^–6^	Mean (low level) reflectivity of bitumen
Collection by a 50 mm diameter lens at 200 mm distance, normal to the surface (Ω = 0.05 sr)			
Light reaching lens from Lambertian reflection	1.5%	1.2 × 10^–7^	Gold coated off-axis parabolic lenses are available
Change in reflected power for a change in *A* of 0.01AU	2.3%	2.7 × 10^–9^	At a mean absorbance of 1.6 AU
Noise equivalent power (NEP) of detector		2.8 × 10^–10^	D* 5 × 10^8^ cm Hz^1/2^ W^−1^, 2 mm × 2 mm^48^, integration period 0.5s

The level of specular reflection from bitumen depends on the material’s refractive index and the polarisation state of the incident light, via the Fresnel equations. For bitumen, a refractive index (1.575) is assumed, equal to the mean of different indices as measured by Lyne et al*.* at 633 nm^49^. (Material dispersion would likely mean this is reduced slightly at 6 μm and 3.8 μm compared with 633 nm, however there are no reports of longer wavelength measurements that would enable an estimate.) For light polarised parallel to the plane of incidence (denoted ∥), the reflectance would be 3.2%, and for light polarised perpendicular to the plane of incidence (denoted ⊥), the reflectance would be 7.1%. Deliberate control of the polarisation state of the incident beam might therefore be used to either enhance or suppress specular reflections, and both extremes have been included in Table 6.

Comparison of the results in Tables 6 and 7 reveals that both specular and diffuse reflections should be detectable with a good signal to noise ratio (SNR) using a high-quality, cooled detector. The estimated SNR is between 800 and 1800 for the specular reflection and around 10 for the diffuse reflection. The specular reflection is therefore expected to be over 80 times as bright as a diffuse reflection from the same area. As the surface is granular, portions of the surface might reflect in a specular fashion towards the lens, even if the gross angles of incidence and reflection are not equal.

This raises the question of whether specular reflections are desirable or undesirable. The spectroscopic basis for the measurement (Fig. 2) was taken using the diffuse reflection, with optics that rejected specular reflections. The hand-held FTIR spectrometer that took such measurements was only able to take readings from surfaces that were macroscopically flat, therefore flat portions of the surface had to be selected if, for example, making measurements on a granular surface such as asphalt^3^. Therefore there is little information about what the relative proportions of specularly and diffusely reflected light might be, and what a mixed spectrum might look like.

Vanier et al*.* determined the optimum collection geometry for minerals via experimentation, while noting that in the field, the surface would present itself at a range of different angles^34^. They noted that although many spectral features seemed enhanced for specular measurements, those that distinguished different materials were degraded (though preserved) if the measurement was mainly specular.

The refractive index change experienced by the light on hitting the surface (with minimal penetration) will give rise to a specular refection from a smooth surface, but could also result in surface light scattering if the surface is rough, and a diffuse reflection. Light scattering from the bulk of the material, which will have experienced greater penetration and a greater influence of spectroscopic absorption features, will give rise to diffuse reflection. New bitumen samples are smooth and collecting diffusely reflected light from them is difficult. But light collected by our FTIR spectrometer from older samples, which are rougher, may have included both surface reflections and bulk reflections.

There remains considerable uncertainty about the optimum measurement geometry for a spectroscopic measurement, which could be resolved through practical experiments with real asphalt samples. If it is desirable to maximise the signal from diffuse reflections (as was done for previously collected DRIFTS spectra^3^ and in the results in Fig. 2 taken using a hand-held FTIR), it is necessary to suppress specular reflection. Any specular reflection created by a shiny surface could be difficult to suppress and may dominate the signal. Asphalt is not perfectly smooth, but the majority of the surface is horizontal. Therefore, maximum suppression is likely to be achieved by using a large angle of incidence, assuming the collection angle for diffuse refection is *θ*_col_ = 0.

### Parallax management

Because the working specification dictates off-axis light collection, parallax will require management. The distance from instrument to surface is expected to change during the measurement and this will displace the apparent illuminated spot from the detector’s point of view. One way of managing this is to ensure that area from which the light can be detected, the detectable spot, is larger than the illuminated spot, allowing the latter to move about, within limits dictated by the size of the detector. This will also mitigate the likely case that collimated laser beams are not perfectly aligned. Even with perfect optics and co-linear beams, the divergence angle and/or focal area of each beam is likely to be different since these quantities are partly a function of wavelength.

#### Étendue calculation

**Fig. 7:**
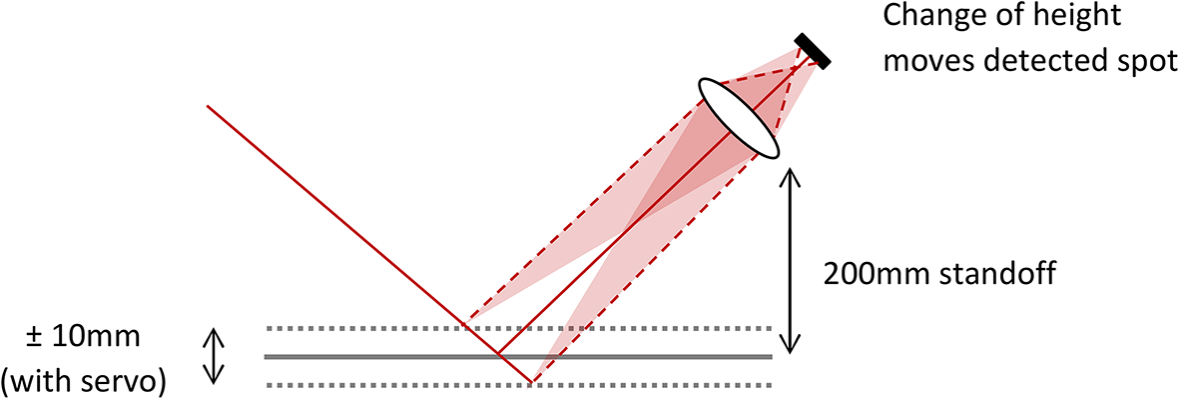
If the surface height changes, a parallax effect causes movement of the detected spot on the photodetector. To detect all laser beams equally, the photodetector must be larger than the extent of the movement imaged at its location.

Variability in the standoff height could mean that the reflected light misses the detector, unless appropriately designed. The problem of parallax in the collection optics is illustrated in Fig. 7. It can be mitigated by increasing the detectable spot and ensuring that the incident laser beam is aligned to the centre of that spot.

The size of the detectable spot is defined by the system étendue at each part of the system, as calculated in Table 8. The magnitude of the étendue is calculated as *b* sin*θ*, where *b* is given by the lateral size of the detected spot on the ground and *θ* by the lens aperture (50 mm diameter). To ensure the spot is always detectable, this étendue must be maintained at the detector, where now *b* is the size of the detector and *θ* is defined by the lens focal length.

**Table 8:** Étendue calculation for detectable spot sizes at 30° incidence, 30° collection, with a 50 mm, high NA collection lens.

	No servo control (± 50 mm)		With servo control (± 10 mm)	
	Detectable spot at target surface	Image on detector	Detectable spot at target surface	Image on detector
Lateral size of detectable spot, *b*	± 50 mm		± 10 mm	
Distance from lens	280 mm	Equal to focal length	280 mm	Equal to focal length
Angular aperture, *θ*_col_ (half angle)	5.1°	27°	5.1°	27°
Étendue = *b* sin*θ*	4.4 mm	≥ 4.4 mm	0.89 mm	≥ 4.4 mm
Required detector size = $\frac{{\acute{e} }\text{tendue}}{{\text{sin}}\theta }$		± 9.8 mm		± 2.0 mm

The analysis of Table 8 reveals that with a 50 mm diameter collecting lens at 45° incidence, parallax can be mitigated by a combination of servo control (bringing the standoff variability to ± 10 mm) and a larger detector area. Using a larger area detector would also provide some alignment tolerance, especially for beams that might not be perfectly co-linear or (more likely) have perfectly similar divergences. High quality mid IR detectors are available in diameters up to 2 mm at these wavelengths; unfortunately, this rules out operation without a servo unless the detection numerical aperture is reduced. Parallax would still be an issue with a 2 mm sized detector however, at these angles.

As shown in Table 9, use of a diffuse collection geometry aligned to the normal has reduced the parallax issues, but still places challenging demands on the detector size, even with servo control.

**Table 9:** Étendue calculation for detectable spot sizes at 30° incidence, 0° collection, with a 50 mm collection lens.

	No servo control (± 50 mm)		With servo control (± 10 mm)	
	Detectable spot at target surface	Image on detector	Detectable spot at target surface	Image on detector
Lateral size of detectable spot, *b*	± 29 mm		± 5.8 mm	
Distance from lens	200 mm	Equal to focal length	200 mm	Equal to focal length
Angular aperture, *θ*_col_ (half angle)	7.1°	27°	7.1°	27°
Étendue = *b* sin*θ*	3.6 mm	≥ 3.6 mm	0.71 mm	≥ 0.71 mm
Required detector size = $\frac{{\acute{e} }\text{tendue}}{{\text{sin}}\theta }$		± 7.9 mm		± 1.6 mm

Table 10 shows that using a narrower (15°) angle of incidence reduces the parallax significantly, such that with servo control, a detector of 2 mm size would be able to collect light from the surface when moving up and down by ± 10 mm, with a small margin for alignment.

**Table 10:** Étendue calculation for detectable spot sizes at 15° incidence, 0° collection, with a 50 mm collection lens.

	No servo control (± 50 mm)		With servo control (± 10 mm)	
	Detectable spot at target surface	Image on detector	Detectable spot at target surface	Image on detector
Lateral size of detectable spot, *b*	± 13 mm		± 2.7 mm	
Distance from lens	200 mm	Equal to focal length	200 mm	Equal to focal length
Angular aperture, *θ* (half angle)	7.1°	27°	7.1°	27°
Étendue = *b* sin*θ*	1.6 mm	≥ 1.6 mm	0.33 mm	≥ 0.33 mm
Required detector size = $\frac{{\acute{e} }\text{tendue}}{{\text{sin}}\theta }$		± 3.5 mm		± 0.73 mm

An alternative mitigation would be to spread the incident laser beam to a larger diameter spot and maintain a smaller detectable spot. This would result in a loss of light. A further disadvantage of this scheme is that it would require the relative intensities of the different beams to be identical (to within 1%) across the whole detectable light field, which might be achievable in principle using suitable optics but could be impractical to achieve. Laser emission is often astigmatic and collimated beams elliptical. Even if the beams have been collimated using identical optics, their propagation, especially divergence, is a function of wavelength and the design proposes significantly different wavelengths for reference and measurement. The result is that for different lasers, the collimated beam profile and its evolution with height is highly likely to be different from device to device and asymmetrical, such that beam profiles may not match to the required degree.

#### Conclusion to parallax design constraints

A large detector (2 mm × 2 mm) combined with a small (15°) angle of incidence would allow mitigation of parallax and some flexibility to accommodate both slight misalignment of collimated laser beams and a finite size of focal spot. Such large detectors are commercially available in a TEC-cooled format. However, there is a trade-off here. “Standoff geometry” section concluded that some separation of specular and diffuse reflections could be desirable, and that this might be achieved by normal light collection combined with a large angle of incidence. Experimental trials will be needed to determine the best compromise.

### Speckle noise

The characteristic dimension, *s,* for subjective speckle (speckles collected using a lens) is given by^50^

16 $$s\hspace{0.33em}\hspace{0.33em}=\hspace{0.33em}\hspace{0.33em}1.2\lambda \frac{f}{a},$$

where *λ* is the wavelength, *f* is the effective focal length of the lens and *a* is the effective aperture radius. We require the use of high NA collection lenses, such that *f*/*a* will take the value of approximately ½. The maximum value of speckle noise, Δ*I*_speckle_, is then given by

17 $$\frac{\Delta {I}_{speckle}}{{I}_{0}}\hspace{0.33em}\hspace{0.33em}=\hspace{0.33em}\hspace{0.33em}\frac{s}{D},$$

where *D* is a linear dimension of the detector (in this case, 2 mm) and *I*_0_ is the incident light intensity. The noise under laser wavelength modulation can be lower than this, but we consider here a worst-case analysis. Following averaging over *N* independent speckles, the speckle noise reduces as follows,

18 $$\frac{\Delta {I}_{speckle}}{{I}_{0}}\hspace{0.33em}\hspace{0.33em}=\hspace{0.33em}\hspace{0.33em}\frac{s}{D\sqrt{N}}.$$

For measurements made along the roadway, *N* is given by the distance over which averaging takes place divided by the distance across the detectable spot.

Values of speckle noise are calculated for the different wavelength regimes in Table 11. From this, we conclude that speckle noise is approx. ¼ of the required limit of detection for the measurement for a capture of spectra from a single snapshot measurement of road surface, and also substantially reduced by averaging along a 10 m section.

**Table 11:** Calculated speckle noise in each wavelength regime.

Parameter	Reference region: 3.8 μm	Carbonyl region: 6 μm
Speckle size *s*	11 μm	18 μm
Speckle noise Δ*I*_speckle_/*I*_0_	5.7 × 10^–3^	9.0 × 10^–3^
Reduced speckle noise for a 5 mm illuminated spot averaged over a 10 m section of road (N = 10,000)	5.7 × 10^–5^	9.0 × 10^–5^
Detected signal for 0.01AU, as Δ*I*/*I*_0_	23 × 10^–3^	23 × 10^–3^

### Summary and system architecture

To summarise this section, the following design decisions can be taken forward.

- Lasers that measure the carbonyl feature have been chosen to operate within specific windows where absorption by water vapour should be minimised. Further mitigation will be needed to reduce the residual effects of light absorption by water at these wavelengths. The reference laser should be unaffected by water vapour.
- Standoff geometry is viable for collection of light from both specular and diffuse reflections with signal to noise ratios that are good and acceptable respectively. To achieve this demands a collection lens diameter of 50 mm.
- Diffusely reflected signals are maximised for a collection angle of 0° to the normal.
- The choice of angle of incidence will be a compromise between the demands of parallax management, for which an angle close to the normal is preferred, and the requirement to suppress specular reflection, (for which a larger angle of incidence would likely be needed). The balance between these competing priorities can only be determined experimentally, therefore the optical design will need to enable changes to be made flexibly without altering the rest of the system.
- For the largest high-quality detector commercially available in a TEC-cooled format, of size 2 mm × 2 mm, the anticipated effects of parallax can be mitigated at angles of incidence and collection of 15° and 0° respectively.
- For a high NA collection lens and large (2 mm) detector, speckle noise is unlikely to be problematic especially when averaged by movement of the surface with respect to the detector.

**Fig. 8:**
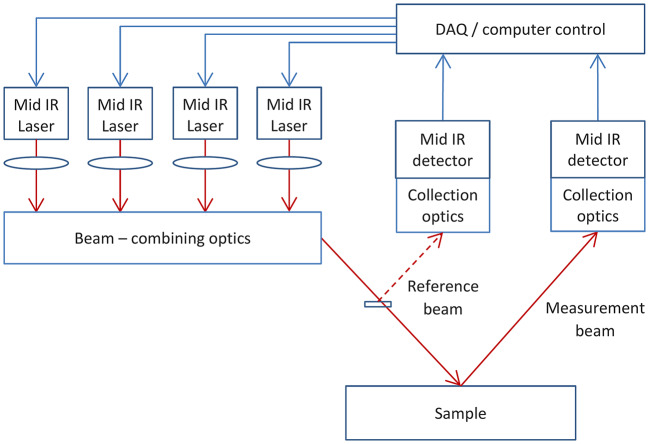
Schematic diagram of concept for instrument for laser-based spectral analysis of roads. (*DAQ* digital acquisition system). Note that reference and measurement beams should be pathlength-matched.

Figure 8 shows a final proposal for system architecture. The proposed signal referencing scheme is explained mathematically in Supplementary Appendix A.

## Conclusion

Mid IR spectroscopy of bitumen has become an established method for laboratory-based analysis of chemical ageing, with a considerable catalogue of reports showing its application to a variety of different types of bitumen and mechanisms of ageing, including field-aged and accelerated ageing. Chemical ageing of bitumen and the resulting onset of embrittlement and loss of adhesion is also considered the primary mechanism leading to loss of aggregate in asphalt. Thus, if the analytical technique could be translated into a field-deployable non-destructive survey method, it could provide a useful enhancement to current survey measurements, especially if the remaining service life of pavement material can be predicted.

To achieve this goal, several fundamental challenges must be addressed.

### Speed

For a measurement to be widely field-deployed, it must be usable on open roads, which for high value roads such as England’s Strategic Road Network (SRN) means operation at traffic speed (any speed up to 50 mph/80 km h^−1^ to enable safe operation in live traffic). Operation at lower speeds should be straightforward for the instrument. Operation at higher speeds would be possible in principle but may require proportionally faster modulation and signal recovery to maintain spectral baselines and signal to noise ratios. Combined with the inherent granularity of asphalt, this translates into a need to acquire spectra quasi-simultaneously at a data rate of 20 kHz. This study proposes a mechanism by which this is achievable, using QCLs operating at discrete, individual wavelength of interest. Ultimately, we believe the design needs additional measurement wavelengths, potentially using QCL arrays to combine beams efficiently, or new developments in dual frequency-comb sources.

### Diffuse reflections

The body of evidence on bitumen spectroscopy and ageing has recently encompassed spectra measured via diffuse reflection^3^, showing that this geometry is also capable of providing ageing information. These measured spectra were the starting point of this study. Growth of C=O bonds is measurable within the 1760–1620 cm^−1^ region. A spectral reference measurement is available at 2633 cm^−1^, which would compensate for changes to the overall optical efficiency of the system.

### Standoff operation

Deployment at speed also means providing the clearance for an instrument to survive changes in road surface height and associated bumps. A standoff distance of 20 cm is proposed, and calculations show here that signal to noise ratios using commercially available optics and detectors are high enough to enable a useful limit of detection. We considered various geometries, concluding that there is a trade-off between the potential need to suppress specular reflection and the consequences of parallax, which can be resolved by experimentation.

### Water vapour

This work shows that judicious choice and tuning of QCLs, combined with a pathlength-matched reference beam, would compensate for the variable and strong absorption by water vapour across the 1760–1620 cm^−1^ region. This is a fundamental constraint, since the C=O bond absorbs strongly here and not elsewhere. Few spectroscopists would choose to operate within this region without the use of dry purge gases, however this work shows, at least theoretically, that it can be done.

### Choice of measurement wavelengths

To fully quantify ageing in a way that is analogous to laboratory measurements would require a light source that combines high spectral brightness (such as a laser) with full spectral coverage as well as a spectroscopic method with high resolution (< 0.3 cm^−1^) to enable operation within narrow spectroscopic “water windows”. Current technology enables measurements to be made at separate wavelengths using discrete mid-IR lasers. Further development of high-resolution laser spectroscopy is needed for greater spectroscopic coverage. With the current system, expansion to other absorption bands (for example to study sulfoxides) would be possible using additional discrete lasers.

Even without full spectra, the proposed instrument would have the ability to measure changes that are associated with ageing via oxidation. By combining this with knowledge of the type of asphalt laid on different sections of pavement, establishing ageing profiles and possibly including trends measured over time, it may be possible to infer ageing characteristics and remaining asset life.

These proposed solutions therefore take measurements a significant step closer to development of a field-deployable instrument for asphalt ageing. Part 2 of this work^12^ demonstrates experimental proof of concept with a new field-deployable instrument. This work could have important consequences for asphalt surveys and for other standoff spectroscopic measurements within the troublesome, water-absorbing region of the mid-IR. A standoff instrument designed for traffic speed could provide spectroscopic data from roads over an entire network, enabling sampling of a far greater proportion of in-service asphalt than is currently available from core samples. Given the considerable body of evidence regarding IR spectroscopic measures of ageing in both laboratory and field-aged samples, it could also provide a useful scientific link between research studies and real-world conditions.

## Supplementary Information

Supplementary Information.

## Data Availability

Data supporting this study are openly available from the Cranfield Online Research Database (CORD), at 10.57996/cran.ceres-2639.
