# Supplementary material for: Mid-IR standoff measurement of ageing-related spectroscopic changes in bitumen in the 6 µm (1700 cm−1) region. Part 1: Measurement strategy and instrument design principles
Source: Sci Rep. 2025 Jul 11;15:25041. doi: 10.1038/s41598-025-01135-7 (PMC12254496; doi:10.1038/s41598-025-01135-7)
Supplement: Supplementary file 1 — Supplementary Information. [file 41598_2025_1135_MOESM1_ESM.pdf]

Appendix A. Referencing strategy

For the system in Figure 8, the following strategy for measurement referencing is proposed.

Let the measured light intensity for the reference beam I_R at a general wavelength λ be

$$I_R(\lambda, L_R) = I_0(\lambda) \cdot T_{opt}(\lambda) \cdot T_{H2O}(\lambda, L_R) \cdot \ T_{pathR}(\lambda) \quad \text{(A.1)}$$

Where I_0 is the intensity emitted from the laser, T_{opt} is the proportional transmission of the optical elements up to and including the beamsplitter, T_{H2O} is the transmission of water vapour and T_{pathR} is the transmission of the optical elements along the path from the beamsplitter to the reference detector, and the subscript R denotes the reference pathlength. The transmission of water vapour over a pathlength L_R is given by reference to equation (2) such that

$$T_{H2O}(\lambda, L_R) = 10^{-\varepsilon(\lambda) \cdot L_R \cdot C} \quad \text{(A.2)}$$

where C is the average concentration of the water vapour in air along the optical path. For the reference wavelength, denoted λ_0, we know that $T_{H2O}(\lambda_0, L_R) \approx 1$ (ie there is negligible absorption by water vapour) so we have

$$I_R(\lambda_0, L_R) = I_0(\lambda_0) \cdot T_{opt}(\lambda_0) \cdot \ T_{pathR}(\lambda_0) \quad \text{(A.3)}$$

The expression for the recovered intensity in the measurement beam (which strikes the asphalt) is similar, with additional terms representing the effect of the asphalt uncooperative target.

$$I_M(\lambda, L_R) = I_0(\lambda) \cdot T_{opt}(\lambda) \cdot T_{H2O}(\lambda, L_M) \cdot R_{geom}(\lambda) \cdot R_{asphalt}(\lambda) \cdot \ T_{pathM}(\lambda) \quad \text{(A.4)}$$

R_{geom} is the bulk geometric reflectance of the surface according to equation (15), which is a function of the instrument height and tilt, and $R_{asphalt}$ is the diffuse spectroscopic reflectance of the asphalt material, for example as illustrated in Figure 2. For the reference wavelength λ_0, similarly we have

$$I_M(\lambda_0, L_R) = I_0(\lambda_0) \cdot T_{opt}(\lambda_0) \cdot R_{geom}(\lambda_0) \cdot R_{asphalt}(\lambda_0) \cdot \ T_{pathM}(\lambda_0) \quad \text{(A.5)}$$

Forming a ratio between the recovered signals in both beams at each wavelength gives

$$\frac{I_M(\lambda, L_R)}{I_R(\lambda, L_R)} = \frac{I_0(\lambda) \cdot T_{opt}(\lambda)}{I_0(\lambda) \cdot T_{opt}(\lambda)} \cdot \frac{T_{H2O}(\lambda, L_M)}{T_{H2O}(\lambda, L_R)} \cdot R_{geom}(\lambda) \cdot R_{asphalt}(\lambda) \cdot \frac{T_{pathM}(\lambda)}{T_{pathR}(\lambda)} \quad \text{(A.6)}$$

The first terms cancel exactly, giving

$$\frac{I_M(\lambda, L_R)}{I_R(\lambda, L_R)} = \frac{T_{H2O}(\lambda, L_M)}{T_{H2O}(\lambda, L_R)} \cdot R_{geom}(\lambda) \cdot R_{asphalt}(\lambda) \cdot \frac{T_{pathM}(\lambda)}{T_{pathR}(\lambda)} \quad \text{(A.7)}$$

Similarly for the reference wavelength λ_0 we have

$$\frac{I_M(\lambda_0, L_R)}{I_R(\lambda_0, L_R)} = R_{geom}(\lambda_0) \cdot R_{asphalt}(\lambda_0) \cdot \frac{T_{pathM}(\lambda_0)}{T_{pathR}(\lambda_0)} \quad \text{(A.8)}$$

Now we form a ratio between the values of equations (A.7) and (A.8).

$$\frac{I_M(\lambda, L_R)}{I_R(\lambda, L_R)} \cdot \frac{I_R(\lambda_0, L_R)}{I_M(\lambda_0, L_R)} = \frac{T_{H2O}(\lambda, L_M)}{T_{H2O}(\lambda, L_R)} \cdot \frac{R_{geom}(\lambda)}{R_{geom}(\lambda_0)} \cdot \frac{R_{asphalt}(\lambda)}{R_{asphalt}(\lambda_0)} \cdot \frac{T_{pathM}(\lambda)}{T_{pathM}(\lambda_0)} \cdot \frac{T_{pathR}(\lambda_0)}{T_{pathR}(\lambda)} \quad \text{(A.9)}$$

A simplifying assumption is made, that the R_{geom} values are equal for the two different wavelengths λ and λ_0. This accounts for the bulk effects on the diffuse reflection of instrument height and tilt

relative to the surface, and also the potential addition of any specular reflection that may be mixed into the signal. It will not account for any wavelength-dependent scattering effects, for example Rayleigh scattering, however it is assumed that such effects will be small compared to bulk changes. That yields

$$\frac{I_M(\lambda,L_R)}{I_R(\lambda,L_R)} \cdot \frac{I_R(\lambda_0,L_R)}{I_M(\lambda_0,L_R)} = \frac{T_{H2O}(\lambda,L_M)}{T_{H2O}(\lambda,L_R)} \cdot \frac{R_{asphalt}(\lambda)}{R_{asphalt}(\lambda_0)} \cdot \frac{T_{pathM}(\lambda)}{T_{pathM}(\lambda_0)} \cdot \frac{T_{pathR}(\lambda_0)}{T_{pathR}(\lambda)} \quad \text{(A.10)}$$

Now we set the final two terms equal to a constant value (termed k). This means any degradation in the optics for each of the measurement and reference paths, caused for example by the appearance of dirt, is not expected to be wavelength-dependent over the course of a measurement campaign. Its value can be checked by performing a calibration check using a known reflector, such as a calibrated coupon of diffusely reflecting gold, at both the start and end of a measurement campaign. The expression simplifies to

$$\frac{I_M(\lambda,L_R)}{I_R(\lambda,L_R)} \cdot \frac{I_R(\lambda_0,L_R)}{I_M(\lambda_0,L_R)} = \frac{T_{H2O}(\lambda,L_M)}{T_{H2O}(\lambda,L_R)} \cdot \frac{R_{asphalt}(\lambda)}{R_{asphalt}(\lambda_0)} \cdot k \quad \text{(A.11)}$$

Expanding the expression for the transmission through water vapour,

$$\frac{I_M(\lambda,L_R)}{I_R(\lambda,L_R)} \cdot \frac{I_R(\lambda_0,L_R)}{I_M(\lambda_0,L_R)} = \frac{10^{-(\varepsilon(\lambda)L_M C_M)}}{10^{-(\varepsilon(\lambda)L_R C_R)}} \cdot \frac{R_{asphalt}(\lambda)}{R_{asphalt}(\lambda_0)} \cdot k \quad \text{(A.12)}$$

where $\varepsilon(\lambda)$ is the wavelength-dependent absorptivity of water vapour, and C_M and C_R are the concentrations of water vapour in the air along the measurement and reference paths respectively. If it is assumed that the air is well-mixed such that C_M=C_R=C, we have

$$\frac{I_M(\lambda,L_R)}{I_R(\lambda,L_R)} \cdot \frac{I_R(\lambda_0,L_R)}{I_M(\lambda_0,L_R)} = k \cdot \frac{R_{asphalt}(\lambda)}{R_{asphalt}(\lambda_0)} \cdot 10^{-(\varepsilon(\lambda)C(L_M-L_R))} \quad \text{(A.13)}$$

For a well-balanced reference beam, L_M-L_R = ΔL is small, and equal to the pathlength imbalance caused by variation in the height of the instrument as it travels along the road. The final expression is

$$\frac{I_M(\lambda,L_R)}{I_R(\lambda,L_R)} \cdot \frac{I_R(\lambda_0,L_R)}{I_M(\lambda_0,L_R)} = k \cdot \frac{R_{asphalt}(\lambda)}{R_{asphalt}(\lambda_0)} \cdot 10^{-(\varepsilon(\lambda)\, C\, \Delta L)} \quad \text{(A.14)}$$

where the final term adds a small error, quantified in Figure 4. Thus, forming the ratios of the measured values of I_M and I_R, at the target and reference wavelengths λ and λ_0, allows cancellation of most of the factors that might disrupt our measurement. This leaves the reflectivity of asphalt at each of the measurement wavelengths λ, normalised by the reference wavelength λ_0. Since the reflectivity at λ_0 is not expected to be affected by ageing, and those at λ are, this measurement can therefore provide an ageing indicator for the asphalt concerned.
